# Business Education for Professional Men

**Published:** 1895-11

**Authors:** C. B. Blackmarr

**Affiliations:** Jackson, Mich.


### Business Education for Professional Men.

BY C. B. BLACKMARR, D.D.S., JACKSON, MICH.

Abstract of a paper read at the Tri-State Dental Meeting, June, 1895.

Professional men usually do everything they have to do in a very unbusiness-like manner. Business education for professional men. Do they need it? I think they do. Whenever you see a successful professional man, you will also notice that all his matters are attended to with promptness and dispatch. One thing I have noticed in my practice is that patients who have the most to do and those who do the most business are those who keep their appointments the sharpest.

A lazy or slow professional man usually thinks because he has or takes plenty of time his patients and every one else has.

The law of supply and demand will work just as well in professional work as in any other. If a dentist's patients want his services at ten dollars an hour, he has a right to ask it, because his time is worth it. But a man whose time is not worth it has no right to charge it, and his patients will resent it, and his practice in time will show the results of such dishonesty.

A man's patients are usually willing to pay him what that man's time is worth and no more. And just because a recent graduate happens to hear of a certain professor getting $50 for looking at a tooth is no reason why he should try to get $50 for consultation fee.

I once heard a successful business man ask a recent graduate this question: "How do you know what to charge a patient when you have finished a filling?" "I don't know," said the graduate. "Don't you have some basis upon which to calculate what your fees should be?" "No, sir," said the graduate. "Can't you charge for your time by the hour or some such way?" "No, sir. But I know of a practitioner who stutters and when asked how much a certain piece of dental work would cost, says f—f—f, and if the patient looks scared, he says *forty*; but if he does not flinch, he says *fifty* dollars, and I guess I will do that way." "All right," said the business man, "but I am glad that I am not obliged to conduct my business on such principles."

I think generally young men, as they are sent to colleges, are impressed with the idea by others that if they only get their professional education, that is all they have to do. So many young men think that their education alone should bring them patients without any further exertion on their part.

I have several young men on my mind now who have not succeeded in their profession, in a financial way at least, owing entirely to their unbusiness-like manner of conducting their practice.

One, for instance, could never be found in his office during the hours stated on his door. Neither was there any word left where he was nor when he would return.

Another young man agreed on his office door to open at 9 A. M. He never was known to keep his agreement. Only a good guesser could tell why. I often wish that that young man would notice whether business places, like banks, etc., opened any more regularly. If that young man ever made an appointment with a patient, it would usually be to come in some afternoon next week, or if he did actually mention a day and time, he always kept his patient waiting half an hour or so. The patient, of course, not keeping the next appointment so closely.

A man who won't keep his appointments in one thing won't in others. If he don't keep his office hours, he won't pay his debts when he says he will. A man who is not honest enough to keep appointments will not fill a tooth nor do anything else honestly.

I have gone into professional men's offices during office hours and found the reception room open, vacant and quiet as death. No one to attend to whoever happens in, nor to tell when the professional man would be in.

How long do you think a bank or business place would exist upon such principles?

A professional man suffers proportionately as much. A traveling dental agent once told me that he went into one of the most elegantly furnished dental offices in this State, and waited forty-five minutes without being waited upon by any one. He knew some one was in the laboratory or operating room, because he could hear them talking. Patients came in and would not wait so long, and went away disgusted. That dentist quit practicing on account of lack of patients in a few years.

Patients often blame dentists for not doing good work for them, when the patients themselves are to blame. Dentists often blame their patients as an excuse for not doing good work for them, when the patients would have gladly given the dentist more time and larger fees, if they had known they would have had better work done by so doing.

The dentist and patient are both, many times, to blame for low-grade work. The class of patients a dentist has should decide the class of work he does. If he would like to do another class of work from what he is doing, he must work into that class, letting go the other class gradually.

A professional man, to be financially successful, must please the patients he has, expects to have, or wants to have. He must satisfy them in his promptness, his fees, in his operations, in his appearance, manner, etc. Professional men and their methods are a continual puzzle to the business men. Their basis of professional fees is a query to them. How a physician or dentist has a right to treat abscesses, tumors, etc., mouth in and mouth out, with no apparent benefit, and to charge for the same, is a mystery to business men.

These subjects should be studied out and shown to the young professional men, so they could answer intelligently and in a prompt manner all such questions when asked by their patients, or any one else.

